# Predicting protein functions by relaxation labelling protein interaction network

**DOI:** 10.1186/1471-2105-11-S1-S64

**Published:** 2010-01-18

**Authors:** Pingzhao Hu, Hui Jiang, Andrew Emili

**Affiliations:** 1 Department of Computer Science and Engineering, York University, ON, Toronto, Canada; 2 Donnelly Centre for Cellular and Biomolecular Research, University of Toronto, ON, Toronto, Canada

## Abstract

**Background:**

One of key issues in the post-genomic era is to assign functions to uncharacterized proteins. Since proteins seldom act alone; rather, they must interact with other biomolecular units to execute their functions. Thus, the functions of unknown proteins may be discovered through studying their interactions with proteins having known functions. Although many approaches have been developed for this purpose, one of main limitations in most of these methods is that the dependence among functional terms has not been taken into account.

**Results:**

We developed a new network-based protein function prediction method which combines the likelihood scores of local classifiers with a relaxation labelling technique. The framework can incorporate the inter-relationship among functional labels into the function prediction procedure and allow us to efficiently discover relevant non-local dependence. We evaluated the performance of the new method with one other representative network-based function prediction method using E. coli protein functional association networks.

**Conclusion:**

Our results showed that the new method has better prediction performance than the previous method. The better predictive power of our method gives new insights about the importance of the dependence between functional terms in protein functional prediction.

## Background

Currently the sequencing of many genomes has brought to light the discovery of thousands of possible open reading frames which are all potentially transcribed and translated into gene products. For many proteins, little is known beyond their sequences, and for the typical proteome, between one-third and one-half of its proteins remains uncharacterized. For example, yet despite being the most highly studied model bacterium, a recent comprehensive community annotation effort for the fully sequenced reference K-12 laboratory strains indicated that only half (~54%) of the protein-coding gene products of *E. coli *currently have experimental evidence indicative of a biological role [1]. The remaining genes have either only generic, homology-derived functional attributes (e.g. 'predicted DNA-binding') or no discernable physiological significance. Moreover, as we know that proteins play role in many biological processes, due to the complexity of biological systems, so many functions of these proteins may have undiscovered yet. As a result, a major challenge in modern biology is to develop methods for determining protein function at the genomic scale [2].

It is widely known that proteins seldom act alone; rather, they must interact with other biomolecular units to execute their function. Protein-protein interactions operate at almost every level of cellular functions [3]. Thus, implications about function can often be made via protein-protein interaction studies. These inferences are based on the premise that the function of unknown proteins may be discovered through studying their interaction with a known protein target having a known function. It has been postulated that a far better way to systematically uncover gene function and the higher-level organization of proteins into biological pathways is by the examination of these interaction networks via proteomic, genomic and bioinformatic approaches, which is essential to discovering the biological context of protein functions and the molecular mechanisms of underlying biological processes [2,3].

Basically, there are two types of automatic function prediction paradigms by analyzing the entire set of functional associations recorded between gene products in the context of a network. The first one is to use the set of associations in the network to propagate the functional labels from well characterized protein nodes to those with limited or no annotations. Many functional prediction studies following this paradigm are often focused first on sub-grouping or clustering the interaction networks into functional modules based on the pattern or distribution of protein nodes and interaction links which can be highly suggestive of shared functions. These modules may be distinct or overlapping. Any unannotated gene products in a given module can be subsequently assigned the most common functional annotation(s) associated with its interacting partners or neighbors [2]. This 'unsupervised' approach often works well if there is extensive coherent annotation available and relatively few uncharacterized proteins per cluster, but there can be difficulty if a module contains many proteins without annotations or with diverse, seemingly unrelated functions. Most of the methods for identifying modules operate on the underlying assumption that proteins within modules are more tightly connected than proteins in different modules; Broadly speaking, the clustering methods we consider are either specific to the network domain, or are based on standard distance- or similarity-based clustering techniques; in the latter case, the key issue is typically in deciding on a suitable measure of distance or similarity between two proteins in an interaction network. These methods differ in the extent to whether they use only local neighborhood information when clustering whereas others use more global features of the network.

Alternate computational methods have been devised to automatically assign functional labels, such as gene ontology (GO) terms [4], to the uncharacterized proteins present in an interaction network in a 'supervised' manner according to the annotations of the broader neighborhood of interacting gene products. Differentiating from the module-based methods cited above, these newer approaches often exploit both the global and local properties of network graphs [5,6]. The trade off is that additional error or uncertainty may be introduced by assuming functional similarity among more loosely connected gene products that are more than one step apart in an interaction network.

Supervised computational methods for automatically assigning functional terms to previously uncharacterized genes based on the categorical properties of their annotated interaction partners have been widely developed [5-10]. Schwikowski et al [7] developed majority counting method to predict for a given protein up to three functions that are prevailing among its neighbors. Nabieva et al. [9] proposed the functional flowmethod which simulates a network flow of annotations from annotated proteins to target ones. They formulated the annotation problem as a global optimization problem, where a unique function is assigned to an unknown protein so as to minimize the cost of edges connecting proteins with different assignments. Chua et al. [5] defined the functional similarity between a pair of proteins by taking both the direct and indirect neighbors of the protein pair into account. They showed that level 2 and 3 neighbors have an above average likelihood of sharing functional similarity. A weighted averaging method based on functional similarity weight between the proteins is defined to predict the function using level 1 and level 2 neighbors. It has been shown to outperform some of the existing methods that use interconnection network information in the three main categories of GO. Lee et al. [6] developed a new kernel logistic regression (KLR) method for protein function prediction based on diffusion kernels. In the KLR method, the authors incorporated the correlation among biological functions into their model by identifying a set of functions that are highly correlated with the function of interest using the *χ*^2 ^test. The prediction accuracy is comparable to another protein function classifier based on the support vector machines with a diffusion kernel. Hu et al. [10] proposed a network-based protein function prediction method that assigns functions to unannotated proteins in a functional association network. The algorithm includes two steps: the first step is to compute function affinity (or function profiles) of uncharacterized proteins to the functional categories we are interested using the functional information from their level 1 (direct) neighbors and level 2 (indirect) neighbors; the second step is to evaluate the correlated functional profiles based on a penalized logistic regression model and uses a stepwise variable selection procedure to select optimized function profiles in the final model to estimate probability of uncharacterized proteins assigned to the interested functions. Their experiments showed that their method makes improvements of prediction accuracy compared to existing techniques.

These methods can be roughly categorized as either simple, local, guilt-by-association techniques or global, network optimization procedures. While both approaches often achieve similar performance [11], there are potential deficiencies in the procedures. For example, since functional terms are often interrelated (e.g. the GO hierarchies), the correlation structure of the respective functional categories can potentially be exploited. Hence, to deduce the biological role(s) of a particular protein, one should take into account the full spectrum and relatedness of available annotations of the interaction partners when evaluating a particular functional category.

In this study, we proposed a relaxation labelling method for network-based protein function prediction. We explored to incorporate the inter-relationship among functional labels into the function predictions. The relaxation labelling is employed to iteratively update each node's likelihood for a given function by taking its non-local dependencies into account. The advantages of the method are demonstrated by a recently generated Ecoli protein functional association network [10].

## Methods

### Relaxation labelling method for protein function prediction

In a functional association network, let us assume that *O *is the set {*o*_1_, ..., *o*_*n*_} of *n *proteins to be labelled. *L *is the set {*l*_1_, ..., *l*_*m*_} of *m *possible functional labels (GO terms) for the proteins. Let *P*_*i*_(*l*_*k*_) be the probability that the label *l*_*k *_is the correct label for protein *o*_*i *_and each probability satisfies 0 ≤ *P*_*i*_(*l*_*k*_) ≤ 1 where *P*_*i*_(*l*_*k*_) = 0 implies that label *l*_*k *_is impossible for protein *o*_*i *_and *P*_*i*_(*l*_*k*_) = 1 implies that this labelling is certain.

The relaxation labeling process includes three steps [12]:

1) Initialize the probability *P*_*i*_(*l*_*k*_)^(0) ^of protein *o*_*i *_for function *l*_*k *_. This can be done by assigning an initial and perhaps arbitrary probability for each label and protein. In this study, we used the weighted voting approach as proposed by McDemott et al. [8]. We considered all labelled proteins in the direct neighbors of a given protein *o*_*i *_and calculated the probability *P*_*i*_(*l*_*k*_) ^(0) ^of protein *o*_*i *_for function *l*_*k *_given by:(1)

where *a*_*ij *_is the association score between protein *o*_*i *_and *o*_*j*_. *N *is the set of proteins neighbouring *o*_*i*_. We introduce a discrete value function *θ*(*o*_*j*_, *l*_*k*_), which takes value 1 if protein *o*_*j *_has function *l*_*k *_and 0 otherwise. If protein *o*_*i *_has no neighbours with function *l*_*k*_, we estimate *P*_*i*_(*l*_*k*_)^(0) ^based on the function's prior in GO gold standard we constructed.

2) Update the probability *P*_*i*_(*l*_*k*_) of protein *o*_*i *_for function *l*_*k *_. This is done by considering the probabilities of labels for neighbouring proteins. Let us assume that we have changed all probabilities up to some step, *T*, and we now seek an updated probability for the next step *T *+ 1. We can estimate the change in confidence of *P*_*i *_(*l*_*k*_) by:(2)

where *C*_*ij *_(*l*_*k*_, *l*_*l*_) is the correlation between protein functional labels defined as the conditional probability that protein *o*_*i *_has a label *l*_*k *_given that protein *o*_*j *_has a label *l*_*l*_, *i.e. C*_*ij *_(*l*_*k*_, *l*_*l*_) = *P*(*l*_*k*_|*l*_*l*_). We estimate *C*_*ij*_(*l*_*k*_, *l*_*l*_) based on semantic similarity between functional terms in GO (discussed below). *N *is the set of proteins neighboring *o*_*i*_, and *ω*_*ij *_is a factor that weights the labellings of these neighbours, defined in such a way that . The new probability for label *P*_*i*_(*l*_*k*_) in generation *T *+ 1 can be computed from the values from generation *T *using(3)

**3) **The process of step 2) is repeated until the labelling method converges or stabilises. This occurs when there is little or no change between successive sets of probability values.

### Construct functional association networks

We used an integrated functional association network which merged the physical interaction network and predicted genome context association network [10]. The resulting combined probabilistic network consisted of 80,370 high-confidence (probability >= 75%) putative pairwise interactions encompassing virtually the entire proteome of E. coli, including 4144 proteins.

### Construct GO gold standard

The most widely adopted gold standard system is the Gene Ontology (GO) database [4], which uses a clearly defined, and computationally friendly, vocabulary for representing the cellular, biochemical and physiological roles of gene products in a systematic manner. From the perspective of functional computation, GO provides a standardized way to assess whether a set of genes have similar functions, which has led to its increasing popularity for the many function prediction procedures used in model organism settings [13]. GO terms are organized in a tree-like structure, starting from most general (e.g. biological process) at the root to the most specific at the leaves (e.g. regulation of DNA recombination) distributed across three major semantic domains - molecular function, biological process, and cellular location. Since terms may have more than one parent, they are technically structured as a network called a directed acyclic graph. For instance, "B cell apoptosis" represents a sub-type of both the term "apoptosis" and "B cell homeostasis". Hence, the functional classes are not necessarily independent of one another, and the dependencies are explicitly defined.

While expert curators manually assign terms based on published experimental evidence, most terms are electronically inferred based on sequence similarity to other well-studied gene products or other criteria. Each term is assigned an evidence code stating how the annotation is supported [14], which allows one to assess the reliability of an annotation. If the annotation is based on experimental evidence traceable to an author or publication, it is presumably more reliable than if it was simply inferred through sequence similarity. The GO has over ten such evidence codes, which are not part of the core ontology.

We created the gold standard for the function predictions based on Gene Ontology [4]. In order to have a suitable number of proteins for cross-validation, we only consider those functional categories with the minimum of 30 associated labeled proteins. Since a functional term is too general if it has too many labeled proteins, we also fixe the maximum number of labeled proteins in a function term to be 400. For biological process (BP) terms in the GO hierarchy related to E. Coli proteins [15], we removed the terms that had neither IPI (inferred from protein-protein interaction) nor IGC (inferred from genomic context) evidence codes. We also removed any proteins with NCBI product descriptions as "hypothetical", "predicted" or "putative". The final GO gold standard used in this study included 1444 of the 4144 proteins in the functional interaction network. Since E. Coli proteins have not been well-annotated using GO, there are only 32 GO BP terms which meet the mentioned filtering requirements and are shown in Table 1. A more complete description of the GO gold standard selection can be found in our previous study [10].

**Table 1:** The 32 GO BP terms used in this study

GO_id	GO Description	Number of proteins
GO.0000160	two component signal transduction system phosphorelay	62

GO.0005975	carbohydrate metabolic process	94

GO.0006118	electron transport	143

GO.0006260	DNA replication	43

GO.0006281	DNA repair	59

GO.0006310	DNA recombination	70

GO.0006313	transposition DNA mediated	50

GO.0006350	transcription	205

GO.0006355	regulation of transcription DNA dependent	221

GO.0006412	translation	99

GO.0006508	proteolysis	41

GO.0006811	ion transport	82

GO.0006826	iron ion transport	32

GO.0006865	amino acid transport	49

GO.0006950	response to stress	71

GO.0006974	response to DNA damage stimulus	55

GO.0007047	cell wall organization and biogenesis	43

GO.0007049	cell cycle	48

GO.0007165	signal transduction	35

GO.0008152	metabolic process	320

GO.0008360	regulation of cell shape	35

GO.0008643	carbohydrate transport	75

GO.0008652	amino acid biosynthetic process	93

GO.0009058	biosynthetic process	55

GO.0009103	lipopolysaccharide biosynthetic process	52

GO.0009252	peptidoglycan biosynthetic process	31

	phosphoenolpyruvate dependent sugar	

GO.0009401	phosphotransferase system	39

GO.0015031	protein transport	43

GO.0016310	phosphorylation	31

GO.0045449	regulation of transcription	39

GO.0046677	response to antibiotic	45

GO.0051301	cell division	46

### Measure semantic similarity of functional terms in GO

Different methods have been developed to determine the similarity of two GO terms based on their distances to the closest common ancestor term and/or the annotation statistics of their common ancestor terms [16-18]. There are some drawbacks in these GO semantic similarity measurement methods. For example, Resnik method only considers the information content of a functional term derived from the corpus statistics while the location of a GO term in GO graph is usually ignored. Wang et al. [19] recently proposed a novel method to encode a GO term's semantics (biological meanings) into a numeric value by aggregating the semantic contributions of their ancestor terms (including this specific term) in the GO graph. They designed an algorithm to measure the semantic similarity of GO terms as follows:

Assume that there are two GO terms: *l*_*i *_and *l*_*k*_, which are represented as two directed acyclic graphs (DAGs):  and , respectively, where  is the set of GO terms in , including term *l*_*i *_(*l*_*k*_) and all of its ancestor terms in the GO graph, and  is the set of edges (semantic relations) connecting the GO terms in . The semantic similarity between GO terms *l*_*i *_and *l*_*k *_is defined as(4)

where  and  is the similarity score of GO functional term *q *associated to term *l*_*i *_. Here  is defined as:(5)

where 0 <*r*_*e *_< 1 measures the contribution of edge *e *∈  linking term q with its child term *q'*. *SV*(*l*_*k*_) and  are defined in the same ways as *SV*(*l*_*i*_) and , respectively.

For the 32 GO biological process terms we discussed above, we used the software G-SESAME to calculate Wang's semantic similarity between any pair of GOs [20].

### Performance evaluation

Classifier performance is evaluated by five-fold cross-validation. Briefly speaking, all labelled proteins are arbitrarily split into five representative sub-groups. Each sub-group is kept to have approximately equal number of labelled proteins in each functional GO term. The computational algorithm is then trained on n-1 of the groups, followed by testing on the remaining holdout group. This procedure is repeated 5 times, each time using a different sub-group of gene products as the test set. We quantify the performance of each GO term based on the area-under-the-curve (AUC) in Receiver Operating Characteristic (ROC) curves drawn by plotting sensitivity versus specificity at different thresholds. Each threshold yields one pair of sensitivity and specificity values and, thus, one point on the curve. The single statistic (AUC) provides a quantitative indication of how well a particular functional classifier performs.

To evaluate the overall prediction performance of our algorithm, we plot the precision against recall at different thresholds, which can be calculated as follows: Given a threshold, if a protein has predicted probability larger than it in a function category, we assign the function to the protein, so we compute(6)

where *m*_*i *_is the number of predicted annotations for protein i, *n*_*i *_is the number of known annotations for protein *i*, *k*_*i *_is the number of correctly predicted annotations for protein *i*.

## Results and discussion

**Figure 1:**
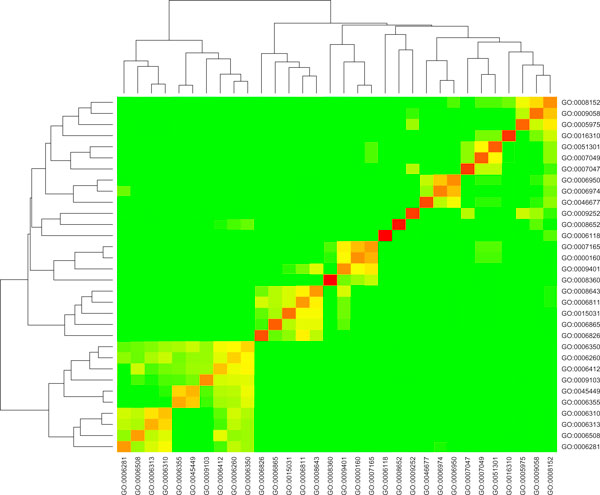
**Heat map shows the semantic similarity among the 32 GO BP terms**. The red colour represents the highest similarity; the green represents the lowest similarity.

Based on the pairwise semantic similarity among the 32 GO terms calculated using formula (4), we made a heat map representing this GO term similarity matrix, which is shown in Figure 1.

As we can see from the plot, some highly correlated GO terms (e.g. GO.0006260.DNA.replication, GO.0006350.transcription, GO.0006412.translation) are grouped into a single block.

**Figure 2:**
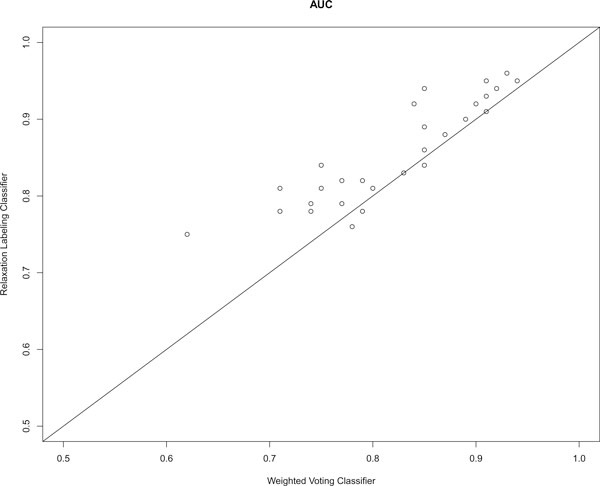
**Relative performance of weighted voting and relaxation labelling classifiers by AUC **. Each circle represents a specific GO BP term.

**Figure 3:**
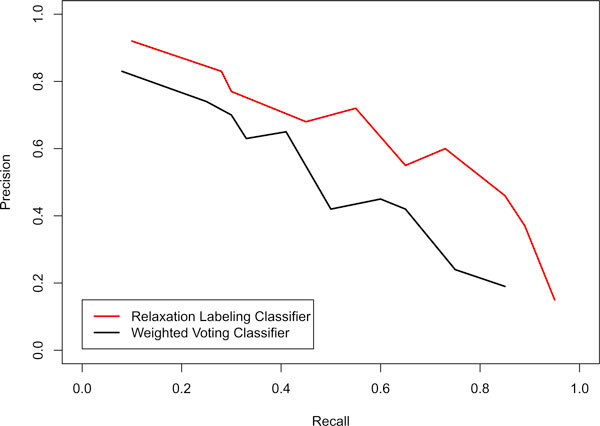
**Precision versus recall of predictions made by weighted voting and relaxation labelling classifiers**.

To compare the performance of the weighted voting and relaxation labelling classifiers, we performed five-fold cross-validation on the built gold standard data. The comparisons of the AUC scores of the 32 GO terms generated by the two classifiers are shown in Figure 2. Overall, the AUC scores for 28 of the 32 GO terms have been increased based on our new function prediction method. The advantage of the new method has been further demonstrated by plotting the recall versus precision curves generated from the predictions based on the two classification methods. As shown in Figure 3, the relaxation labelling based approach has larger recall and precision than those based on the weighted voting approach. We also observed that highly correlated GO terms as shown in Figure 1 have larger prediction improvement than uncorrelated GO terms based on our new method.

In this study, we used the GO structure-based method to quantify the similarity between GO terms and demonstrated its advantage by incorporating this information in E. Coli protein function prediction. We are exploring to compare the performance of this method with information-content-based approaches to measure GO similarity in protein function prediction. In the future, we will apply the new method to protein functional association networks generated in other species and compare its performance with other representative methods in detail.

## Conclusion

In this study, we have proposed a new method for efficiently exploring relevant non-local dependencies of functional labels in the protein function prediction task. Our approach is able to take into account the correlations among GO terms. It includes two major steps: first, initial label assignment is made by a local classifier; second, the dependencies among GO labels are taken into accounted and propagated using an iterative relaxation procedure. The potential trade-off is that additional error or uncertainty may have occasionally been introduced by assuming functional similarity among more loosely connected proteins. We evaluated the performance of the new method with one other representative network-based function prediction method using E. coli protein functional association networks. Our results showed that the new method has better prediction performance than the previous method. The better predictive power of our method gives further insight about the importance of the dependence between functional terms in protein functional prediction.

## Competing interests

The authors declare that they have no competing interests.

## Authors' contributions

PH designed and implemented the algorithms. PH drafted the manuscript. HJ and AE provided helpful suggestions on the algorithm development. AE and HJ monitored the study. All authors read and approved the final manuscript.
